# Missed opportunities in the evaluation of public health interventions: a case study of physical activity programmes

**DOI:** 10.1186/s12889-017-4683-z

**Published:** 2017-08-22

**Authors:** Sarah Hanson, Andy Jones

**Affiliations:** 1 School of Health Sciences, University of East Anglia, Norwich, Norfolk, NR4 7TJ UK; 2 Norwich Medical School, University of East Anglia, Norwich, Norfolk, NR4 7TJ UK

**Keywords:** Evaluation, Physical activity, Public health, Evidence based medicine

## Abstract

**Background:**

Evidence-based approaches are requisite in evaluating public health programmes. Nowhere are they more necessary than physical activity interventions where evidence of effectiveness is often poor, especially within hard to reach groups. Our study reports on the quality of the evaluation of a government funded walking programme in five ‘Walking Cities’ in England. Cities were required to undertake a simple but robust evaluation using the Standard Evaluation Framework (SEF) for physical activity interventions to enable high quality, consistent evaluation. Our aim was not to evaluate the outcomes of this programme but to evaluate whether the evaluation process had been effective in generating new and reliable evidence on intervention design and what had worked in ‘real world’ circumstances.

**Methods:**

Funding applications and final reports produced by the funder and the five walking cities were obtained. These totalled 16 documents which were systematically analysed against the 52 criteria in the SEF. Data were cross checked between the documents at the bid and reporting stage with reference to the SEF guidance notes.

**Results:**

Generally, the SEF reporting requirements were not followed well. The rationale for the interventions was badly described, the target population was not precisely specified, and neither was the method of recruitment. Demographics of individual participants, including socio-economic status were reported poorly, despite being a key criterion for funding.

**Conclusions:**

Our study of the evaluations demonstrated a missed opportunity to confidently establish what worked and what did not work in walking programmes with particular populations. This limited the potential for evidence synthesis and to highlight innovative practice warranting further investigation. Our findings suggest a mandate for evaluability assessment. Used at the planning stage this may have ensured the development of realistic objectives and crucially may have identified innovative practice to implement and evaluate. Logic models may also have helped in the development of the intervention and its means of capturing evidence prior to implementation. It may be that research-practice partnerships between universities and practitioners could enhance this process. A lack of conceptual clarity means that replicability and scaling-up of effective interventions is difficult and the opportunity to learn from failure lost.

## Background

The adoption of an evidence-based approach in the design, delivery and evaluation of public health programmes and interventions is an increasing requirement of programme funding [1]. The scientific principles underpinning this should include reviewing and building a solid evidence base, promoting best practice on effectiveness and implementing evidence based public health policies, all of which are accepted practice in evidence based medicine [2]. However, evidence based medicine is often built on the ‘gold standard’ of design validity in randomised controlled trials. This is rarely an appropriate methodology for multi-faceted, complex public health interventions where there are likely to be a number of interacting components and where contextual relevance is crucial [3, 4].

Evaluation design broadly describes the set of tasks required to systematically examine the effects of a public health intervention [5]. However, it has been suggested that there is often a mismatch between the magnitude and importance of a public health problem and the adequacy of evidence on potential interventions to address it [6]. There is a cost of this inadequacy to public health commissioners and practitioners who need evidence on ‘what works, for whom and in what circumstances?’ when faced with difficult choices in prioritising targeted actions to improve health and reduce inequalities [7, 8]. This problem is particularly pertinent in an era of fiscal restraint. Further, effective evaluation should capture the most promising innovations with high quality evidence of impact in a timescale that is relevant to policy makers [9]. Striking the balance between scientific design and real world pragmatism in intervention design and evaluation thus represents significant challenge. Commensurate with this is the need for an effective tool for the evaluation of public health interventions.

There are many evaluation tools in public health, such as those developed by the World Health Organization for obesity prevention programs [10]. This paper explores one such tool and its use in building evidence in physical activity interventions. It is more than fifty years since Morris and colleagues published their landmark paper showing the link between physical activity at work and heart disease [11]. Since then, physical activity has been shown to have wide ranging long term health benefits and inactivity to make a major contribution to early mortality [12, 13]. What is rather less known is what to do about inactivity in terms of effective health promotion interventions at a population level. Despite population-level programmes such as the UK Change4life [14] there remains a situation where few people are active enough to be of significant benefit to their health, and inactivity levels remain high in both adults and children [15]. One way to increase physical activity is to increase walking. It is a simple activity, can be integrated into everyday life without high costs, and has been shown to be one of the best ways to improve health and achieve recommended daily amounts of physical activity [16, 17]. However, whilst interventions can increase walking time, evidence tends to be of efficacy rather than effectiveness, and the generalisability and sustainability of many interventions consequently remains uncertain [18].

In 2013 the Department of Health (DH) in England invited cities that had previously received funding for cycling interventions to submit proposals for walking interventions [19]. Between 2013 and 2015, £1.2 million was distributed to the five successful ‘Walking Cities’ in England (Birmingham, Cambridge, Leeds and Bradford, Manchester and Norwich). The primary purpose was to get inactive people physically active via a walking intervention with a focus on tackling health inequalities. A summary report of the outcomes can be found on-line [20]. Aside of achieving physical activity outcomes, DH was explicit that funded projects would trial different approaches to generate learning to contribute to Public Health England’s evidence base. To do this cities were expected to undertake a simple but robust evaluation using the Standard Evaluation Framework for physical activity interventions (SEF) [21].

The SEF was developed in 2012 as one of a suite of evaluation frameworks to support and promote high quality consistent evaluation of weight management, diet and physical activity interventions. This was in response to many evaluations being poorly designed, focusing disproportionately on participant satisfaction rather than appropriate measurements such as health outcomes, and with inconsistent reporting making comparison difficult [22]. For example a recent evaluation of a variety of established physical activity interventions in England identified very variable levels of monitoring and evaluation [23]. Subsequent to this, DH’s emphasis on using SEF in the Walking Cities was that it was mandated at the funding stage so that interventions would be effectively monitored and evaluated from the start of the process and thereby help fill evidence gaps about how to get more people walking [21].

Despite the call for an evidence base of innovative physical activity interventions with potential for successful scalability [24, 25], the need continues for effective ways to improve health behaviours, particularly amongst those in lower socio-economic groups [26]. An evidence base of effectiveness can only be populated and deemed reliable if the evaluating tool is capable of, and has been effectively used to, systematically examine the effect of the intervention and its implementation in context. This paper examines the pragmatic reporting of such a tool by practitioners, rather than researchers, under real world circumstances.

It uses data generated by the Walking City initiative in five cities in England as a case study in the use of an evaluation tool to appraise physical activity interventions. The proposals provided by the cities at the application stage and final evaluation reports were analysed against each of the 52 criteria in the evaluation framework that had been mandated by the funder. Our aim was not to evaluate the outcomes of this programme or to be unnecessarily critical of program reporting. Rather, it was to evaluate whether the evaluation process had been effective in generating new and reliable evidence on intervention design and what had worked in ‘real world’ circumstances.

## Methods

### Data

Data are described in Table 1 and primarily consisted of bid documents and end of intervention evaluations provided to DH. This comprised sixteen documents, running to 637 pages. Each city completed a bid document based on a pro-forma which consisted of five sections: description of the proposal, making the strategic fit of the proposal and the rationale, the economic case for funding showing the benefits to the community and expected impact, the implementation and delivery plan, and finally monitoring and evaluation. There was no pro-forma for the final reporting. However, the guidance specifically stated that SEF should be used to demonstrate achievement of physical activity objectives and to generate learning to fill evidence gaps about interventions seeking to get more people walking and more active*.* The authors received all documentation for review from DH in February 2016.

**Table 1:** Walking Cities’ data against which the SEF tool was evaluated

Source	Documents
Department of Health	➢ Invitation to submit proposal for additional revenue funding for walking as a supplement to the Cycle City Ambition Grant (guidance document) ➢ Cycle City Revenue funding for Walking application form (the bid document)
Birmingham City Council	➢ Walking Revolution. Cycle City Revenue funding for Walking bid document ➢ Creating more active communities. Walking cities final report: summary and recommendations (Birmingham, Leeds and Bradford and Norwich combined report by Living Streets^a^)
Cambridgeshire County Council	➢ Walk Local. Cycle City Revenue funding for Walking bid document ➢ End of funding reports from 5 individual projects: Walk Buggy; Walk Health; Walk Work; Walk School; Walk Aware
Leeds City Council and the City of Bradford Metropolitan District Council	➢ Best Foot Forward. Cycle City Revenue funding for Walking bid document ➢ Creating more active communities. Walking cities final report: summary and recommendations (Birmingham, Leeds and Bradford and Norwich combined report by Living Streets^a^)
Greater Manchester authorities	➢ Get Active in Greater Manchester. Cycle City Revenue funding for Walking bid document ➢ Get Active in Greater Manchester evaluation report (general) ➢ Individual evaluations for 8 projects
Norwich City Council	➢ Walk to. Cycle City Revenue funding for Walking bid document ➢ Creating more active communities. Walking cities final report: summary and recommendations (Birmingham, Leeds and Bradford and Norwich combined report by Living Streets^a^) ➢ Walk Norwich Programme Report

^a^Living Streets ran the walking project in 3 of the cities https://www.livingstreets.org.uk/

### Data analysis

Prior to data extraction, clear principles were established. These were that all documentation would be read and analysed systematically against the 52 SEF criteria, with cross checking between the documents at the bid and reporting stage and with reference to the SEF guidance notes. SEF criteria are grouped into seven parts: 1) programme details 2) evaluation details 3) demographics of individual participants 4) baseline data 5) follow up data (impact evaluation) 6) process evaluation 7) analysis and interpretation. Of the 52 criteria in SEF, 34 are listed as essential and 18 as desirable. The full framework is available on-line at http://tinyurl.com/y7vz8yq4. Essential criteria are described as the minimum recommended data and information required to evaluate an intervention and desirable criteria are additional data that would enhance the evaluation. All 16 documents were read and analysed line by line and compared against the SEF criteria. Relevant data were extracted, colour coded for essential or desirable criteria, and managed in Microsoft Excel. So as to ensure as full an understanding of the evaluation as possible, reports with missing data were checked against the original applications for programme details to attempt to determine why this might have been so.

Analysis was led by SH with regular meetings with AJ for cross checking and interpretation of the data. Reporting followed NIHR guidance for organisational case studies using the four domains of describing the design, data collection methods, data analysis, and interpretation of the data to support a clearly structured methodology and reporting process [27].

## Results

A list of the projects delivered by the five cities can be found in Table 2. Interventions in three cities (Birmingham, Leeds and Bradford and Norwich) were co-ordinated by a national charity, Living Streets, and they produced a single report for the three cities. Norwich City Council additionally submitted its own report. Cambridge City Council ran five interventions and submitted five reports. Manchester City Council submitted a general report and a report for eight individual interventions. Some interventions were not evaluable under SEF. Where this was the case, for example a walking festival in Manchester which was essentially a public event, they have not been included in this evaluation. The findings from those interventions which potentially were evaluable under SEF criteria are summarised below and are grouped according to the seven sections of a SEF evaluation.

**Table 2:** Activities described by the five Walking Cities in their final reports

City (project)	Activities described in final report	
Birmingham (Walking Revolution)	Led walks to the park for key stage 2 pupils at one city primary school Pledge cards for individuals Themed walks Community Street Audits Small grants fund Walking Champions Social media work Strategic work to share learning	
Cambridge (Walk Local)	Promotion of Walk4Life and WfH campaigns (various media), maps and community walks. 7 walks with 108 participants Walk to work week activities with 17 employers Health walks in 3 medical centres. 27 participants Beat the Street piloted in 4 primary schools. 890 participants (3 week intervention) Weekly buggy walks / wild play with young parents and children under 5 years of age. 230 attendances	
Leeds and Bradford (Best foot forward)	Themed walks Community Street Audits Small grants fund Tendering delivery to local community organisations Social media work Strategic work to share learning 3 million steps social reward scheme involved 388 people who walked over 15 Million steps in Spring 2015.	
Manchester (Get Active)	Active Oldham Outdoors Project: 26 Level 1 health walks with 143 participants 14 new volunteer walk leaders Capital funding from Public health to improve walking infrastructure in 9 parks Active Trafford Greenspace Project: 20 led walks with 278 participants 23 new volunteer walk leaders 3 GP Surgeries piloting ‘walk prescribing’ or other methods of promoting walking to patients. 3 Workplace Walk led walks events held 5 new Workplace Walks mapped and promoted Bridgewater Canal Walks Project: 22 recreational themed walks with 74 participants Self-led walking trail Partnership working with a community Leisure Trust Four week Walking Festival East Manchester Moving: 23 led walks with 163 participants 10 led cycle rides with 96 participants 8 volunteer action days 24 volunteers trained as volunteer walk leaders	Salford Ranger team project: 1 new self-guided walking route 11 additional recreational walks with 107 participants 12 volunteers trained as walk leaders 12 led cycle rides with 108 participants Stockport Walkaday Walks Project: 13 additional walks with 217 participants 4 new volunteer walk leaders Tameside Active Outdoors Project: 4 new health walks with 30 participants 18 new volunteer walk leaders 1 new patient from new exercise referral programme 5 new self-guided walking routes in production – Proposed launch in 2016 The Green Corridor Project: 9 led health walks with 91 new participants. 5 volunteers trained as walk leaders
Norwich (Walk to)	Group walking champions led walks including 1 km group walk for people with a learning disability Pledge cards for individuals Community Street Audit (1) Small grants fund Beat the Streets project for school children: 1890 participated Social media work Strategic work to share learning Health walks: 185 walks, 12 volunteers and 154 new participants Beat the Street: 1890 participated Travel plans: numbers not reported	

### Programme details

This section consists of 23 questions, of which 16 contain essential criterion, in which details of how and why the intervention is supposed to achieve its objectives and the process for behaviour change should be described. The logic for the interventions or their design was not well described and neither clear rationales, theoretical underpinning nor logic models were supplied for any of the interventions. For example, one of the Manchester schemes stated that the aim was to increase independent walking, another to increase walking rates and a further scheme aimed to increase provision of led works and to integrate green space volunteering opportunities but no further detail was documented to support the rationale of the intervention. Generally, the target population could have been more clearly specified. It was clearly described in one school based intervention in Cambridge with targeted schools and pupils and there were broad descriptions in the other four Cambridge schemes (for example, young parents in the walk buggy scheme; working adults in the walk to work scheme; sedentary adults in the health walk scheme and specific community groups and companies in the walk aware scheme). Norwich, Birmingham, Leeds and Bradford and Manchester specified targeted neighbourhoods of deprivation or low activity. The method of recruitment and referral was not recorded in any of the cities’ evaluation reports. The assessment of unintended consequences was only completed in two of the evaluations (both in Cambridge) despite it being an essential criterial. The relevant policy contexts, health needs assessments and equality impact assessments were not described in the final reports although there were some references to these within the bid documents and the bid outlined the context of the walking intervention. The detailed breakdown of costs for each intervention and the cost per participant were reported for all interventions which ranged from £1800 to £63,000 and £10.87 to £875 respectively. The total cost of the Walking Cities was £1.75 million, which included £1.2 million central funding from DH and the rest from local funding.

### Evaluation details

The way in which the evaluation should be planned to collect and analyse data should be detailed in this section of SEF. Advisory notes suggested a preference for independent evaluation. There are two elements within this section, both essential; the intended type of evaluation and the methods and timing of data collection. Manchester wrote that it would use questionnaires, surveys and case studies, although the tools were not specified. Cambridge and Norwich stated that Intelligent Health would evaluate the use of ‘Beat the Street’ for school children. Birmingham, Leeds and Bradford and Norwich all stated that they would use the ‘Upshot’ monitoring system [28] to collect data on people who received a walking message and how many people actively participated in a walking activity delivered through the project but a tool for assessing physical activity was not specified. Manchester wrote in its bid document that it would work with its academic partner (a local university) but no details of this were given in the final reports. Transport for Manchester commissioned a qualitative report to gain insight into the perceptions and motivations of participants taking part in the walking programmes. The Norwich school-based walking scheme was independently evaluated by the University of East Anglia and is published elsewhere [29]. A process evaluation of the Norwich walking scheme was also independently conducted and is published elsewhere [30].

### Demographics of individual participants

This section has five essential questions to collect individual-level data on participant age, sex, ethnicity, disability and socio-economic status. This was not recorded for the Birmingham and Leeds and Bradford report by Living Streets. The first four of these items were provided by all the Manchester schemes and the Norwich scheme. The Cambridge ‘Beat the Street’ school walking scheme did not record any of this data. Socio-economic status, or a proxy for it, was reported poorly, although all bid documents stated that the interventions would run in areas of known deprivation. The interventions in Manchester used home postcodes to infer consumer classifications and reported the same classifications across their eight interventions [31]. The two desirable criteria to enhance the evaluation or provide an indication of potential confounding factors (such as marital status or medical history, or parental physical activity in the case of the school based intervention) were not reported at all.

### Baseline data and follow-up data (impact evaluation)

The one essential criterion in the baseline and follow up data sections sought the recording of measures of physical activity behaviour at baseline with a minimum of 3 months follow up to enable an assessment of impact. There was mixed reporting of this. Where there were evaluations at baseline and end of intervention these were imprecisely written with only one reference to a validated tool. For example, Manchester grantees wrote that self-reported weekly walking, physical activity and self-rated health would be recorded but were unable to report follow up data due to re-contact problems. The tools used are not described. Cambridge recorded that it used the validated International Physical Activity Questionnaire [32] but reported regional Public Health data for baseline and gave bar graphs for follow up data with no timescale given for the final recording. The Norwich report stated that 50% of participants undertook 30 min or less activity on 3 days or less at baseline but did not state the number of participants and did not appear to have used a validated tool. The Living Streets report for Birmingham, Leeds and Bradford and Norwich did not record baseline or follow up physical activity data. Instead they used ‘Upshot’ [28], a monitoring rather than a physical activity evaluation tool which estimated how people were reached with a walking message, for example.

### Process evaluation

This section required reporting the flow of participants through the intervention to describe what was delivered, any changes to the intervention and any unexpected outcomes. Although the number of participants recruited was reported in 12 of the interventions, the numbers invited, enrolled, screened or attending each session or contact point was not written into any of the grantees reports. There was very limited written description of what was actually delivered and no description of any unexpected outcomes. Five interventions reported positive participant satisfaction. In terms of planning for sustainability, equipment was left at children’s centres for future use in the Cambridge scheme. The Manchester schemes secured an extra six months of funding to be used to identify future funding work with three partners; Manchester City Council, The Ramblers and Walking for Health. Norwich also secured some funding with local partners to continue for 6 months. There were no sustainability plans within the Birmingham or Leeds and Bradford reports.

### Analysis and interpretation

This section contained four sections: summary of results for primary and secondary outcomes over the course of the intervention, details of further analysis with limitations and generalisability, recommendations to future projects, and dissemination of findings. Most likely as a consequence of the very limited baseline and follow-up data there was no reporting of the summary of results and no further analysis was presented for any of the interventions. Recommendations and changes to future projects was an essential criterion. However, there were only recommendations from two schemes (Cambridge and Manchester). For example that GP referrals into walking schemes should be better utilised, longer time scales for projects to be embedded and better business engagement (all Cambridge) and in Manchester for an improvement to the way follow-up information was obtained. There were no written plans for dissemination of learnings from any of the interventions beyond the submission of the reports.

## Discussion

This study evaluated the reported use of an evaluation tool in the ‘real world’. The Walking Cities’ projects were commissioned not only to increase physical activity but also to increase the evidence base in a pragmatic way by being implemented and evaluated by practitioners ‘in the field’. As such this case study provided a particular insight into the difference between efficacy in a research situation and effectiveness trials [9]. The funded cities were expected to use the SEF tool for systematic and detailed description of the intervention to enable replication, evidence synthesis and wider implementation [3], yet it was found that this requirement was not well followed.

Many of the interventions used walking groups in one guise or another. Such groups have been shown to have multiple health benefits [17, 33]. However, how best to organise these for different social groups is not well understood [30, 34]. Our findings that the Walking Cities’ reports lacked specificity means that the programme did not achieve its aim of adding to the evidence base around how these programmes could be best run. This represents a missed opportunity to test ideas or highlight potential ‘best practice’ [35]. Similarly, the method of recruitment and referral was not described in any of the interventions. This observation supports findings from a systematic review that conceptual clarity in recruitment, methods and reporting within walking intervention trials are poorly developed, limiting the impact of interventions to promote walking [36]. Without such clarity, replicability and scaling-up of effective interventions is not possible. More importantly, the opportunity to learn from failure is lost. This may be because of concerns about ‘exposing’ unsuccessful interventions. Yet it is essential that such barriers are overcome to ensure that the discipline of physical activity promotion is better developed [2].

A fundamental limitation was that the target population and population of participants were not precisely specified in either the bid documents or the evaluation reports. For example, Manchester wrote in its bid document that target areas had been identified based on health data available and the Indices of Multiple Deprivation and Norwich wrote that areas were identified through health mapping local demographic information and professional knowledge. The role of Public Health England and local government is to not only maximise the health of their populations but to reduce inequalities [8]. Yet, preventative interventions are known to be socially patterned and more likely to be successful amongst the more affluent, a process which has been coined the ‘inverse prevention law’ [37]. Specifically, the poorest provision of walking group interventions is often in the most deprived areas, and recruitment to walking interventions results in the uptake of mostly white, well-educated, and middle aged women [34, 36]. Without contrary evidence, there is therefore concern that uptake of the Walking Cities’ interventions could have been socially patterned such as to widen health inequity.

The poor description of the rationale for the interventions, and the lack of a stated theoretical basis for any of the interventions or provision of programme logic models to summarise the intervention plan may indicate a lack of readiness on the part of providers for impact and outcome evaluation [38]. The use of an evaluability assessment, also known as exploratory evaluation, at the planning stage may have helped this by determining how ready the intervention was to be evaluated. This is specifically stated in Section 2 of SEF (evaluation details) which only has two criteria, both essential: type of evaluation and evaluation design and methods and timings of data collection. Greater specification of these in initial bid documents may have ensured that realistic objectives were developed and crucially may have helped select innovations [39]. Additionally, the use of logic models, whereby the rationale and outcomes are graphically depicted, can provide stakeholders with something that can be readily understood and modified in an iterative process before an intervention commences. Logic models are viewed as instrumental in explicitly stating assumptions [40]. Leviton gives the analogy that logic models are like a pie crust – ‘made to be broken’ [39, p. 222], suggesting that the whole idea is to provide stakeholders with something they can understand and thus modify in the light of that understanding to get them on the right track. Such processes of preparatory work can increase the relevance and usefulness of evaluations that follow. Evaluability assessment also considers whether outcomes are plausible and achievable in the timeframe and resources available. There is expectation of a degree of common sense in this assessment, as well as reference to the literature and expertise. Hence plausibility analysis is considered critical [39]. It is noteworthy here that the timeframe from the announcement of the funding to the evaluation stage was only two years. A recent process evaluation with stakeholders of the Norwich Walking Champion scheme found this to be an unrealistic timeframe and that a staged funding process over a longer time period would have been more effective [30]. Indeed a recent review of evaluability noted the tendency of new initiatives to be introduced quickly and suggested that rather than testing if programmes ‘work’, evaluative research should test more general theories about how interventions work by aggregating the evidence [41]. The Walking Cities’ teams met on a regular basis and therefore aggregation and synthesis of evidence across the nine programmes that used walking groups presented an opportunity to test theory across a range of contexts and situations.

Generally, the reports were not clearly written or explained. For example, numerical data were often presented in a way that was not appropriate (for example bar graphs and pie charts rather than simple tables or text commentary), the SEF was not followed stage by stage and there was also a use of case studies and pictorial evidence that was not summarised in a way to be useful for building evidence. This may well represent a training need for evaluators, reflecting a recent review on the potential for improving practitioner training and capacity in implementation research in general and for the SEF in particular [42, 43]. Prior training in evaluability assessment may have also benefited the whole programme of interventions [44]. Without it, interventions may not be designed with evaluation in mind at the early design stages. This is particularly important as in England local authorities have a key role in generating their own evidence and sharing lessons learned [8]. An outcomes evaluation and a process evaluation in Norwich with a university partner and a qualitative study with a consultant in Manchester were the only independent evaluations reported. Such partnerships are key to promoting trustworthiness and organisations such as local universities are available for help and advice and to build evidence for wider dissemination [8].

A challenge for any evaluation tool is how to capture the messiness of ‘real world’ whilst having a systematic approach that allows for comparisons across interventions. Whilst structured approaches like the SEF can be of use to those with little experience, the importance of being able to take a more nuanced approach based on judgement and experience has been highlighted by others [41]. Thus there is a need for an approach that balances the need for high quality, robust, realistic and proportionate evidence with the need for innovation when building evidence of the most promising interventions [45].

Throughout the reports there is a real sense of the commitment and enthusiasm for the individual projects and there appears to be examples of potentially interesting and innovative practice listed in each of the cities’ reports. This includes a walk prescribing scheme in general practice in Manchester, a project working in partnership with Bangladeshi, Pakistani and Afro-Caribbean groups in Oldham, and a ‘Park Walk’ put on before a ‘Park Run’ in Norwich. However, because of the lack of detailed reporting that is necessary for those unfamiliar with the projects there was a failure to capture both essential systematic evidence on physical activity improvement and evidence of promising practice. Whilst projects were all described, the explanations lack the level of detail that would give a real feel for the merits of the programmes to an outsider. Many of these projects may have been novel, warranting further investigation. Equally, it may have been that none of the interventions were innovative. Yet the way these are reported, without clear recommendations or practical suggestions for future practice and no dissemination plans may mean that potentially innovative practice remains localised. The challenge therefore is to collate evidence which captures complexity without a narrow, reductionist route [46].

### Strengths and limitations

A strength of this analysis is the access to multiple reports comprising sixteen documents in total, running to 637 pages of evidence which were compiled against a standardised evaluation framework. A further strength is our use of a rigorous framework to extract data from the reports which was subsequently checked by both authors. It should be noted, however, that this study was an evaluation of reporting and there may have been additional evaluation work that was not written into the reports. For example, there may have been longer term academic partnerships that were not discussed which could lead to further publications from evaluation in the future, although our conversations do not indicate this to be the case. A further consideration is that these reports were compiled to meet the needs of funders and although there was an expectation that the findings would add to the evidence base this may not have been the main concern for those writing the reports. Whilst we are not conversant with the context of the individual schemes we can speculate that short time-periods for scheme development, limited staffing capacity and other competing demands in local authorities may well have influenced both the perceived importance and priority given to completion of the SEF document at the end of the funding period*.* Additionally, although the way these reports were communicated may not be ideal for research synthesis they may have met the needs of practitioners who are used to reporting in a different way.

## Conclusions

Decisions on implementing programmes can only be based on the best evidence available at the time, and therefore it is essential that evaluators generate the best, and most robust evidence possible to help build the scientific case on what works and what does not in practice. This is no more apparent than in the rising levels of physical inactivity, coupled with competing financial demands within public health worldwide. Evaluation frameworks allow an understanding of a program’s context and improve how it is conceived and conducted [40]. This case study evaluated the use of one such standardized tool for evaluating physical activity interventions. We found poor adoption of the tool which represented a missed opportunity to appraise multiple physical activity interventions. There is no suggestion of poor intent on the part of those who completed the reports, but our findings suggest a mandate for preparatory evaluability assessment prior to intervention implementation. This does not need to be cumbersome or bureaucratic. Rather, taking the time to develop the intervention on a clear theoretical basis; to establish how evidence will be captured (using a logic model or similar tool) and to appraise the capability and training needs of those evaluating prior to the start of the intervention could yield more robust evidence and ensure that a more nuanced approach is used where it is deemed that full evaluation is not needed. This period of preparatory work could ensure that only appropriate, useful and relevant programmes are evaluated and that those that are evaluated are evaluated well. Where funds are bid for, we would suggest in future that such preparatory work is submitted at the bidding stage rather than at the end of the reporting period. It may be that research-practice partnerships between universities and practitioners could enhance this process due to their independence. This may also increase the likelihood that such ‘real world’ evidence is disseminated and contributes much more fundamentally to the wider field, beyond local reporting.

## Abbreviations

DH: Department of Health
SEF: Standard Evaluation Framework for Physical Activity Interventions
